# Chemical Exchange Saturation Transfer MRI: What Neuro-Oncology Clinicians Need To Know

**DOI:** 10.1177/15330338231208613

**Published:** 2023-10-23

**Authors:** Pejman Jabehdar Maralani, Rachel W. Chan, Wilfred W. Lam, Wendy Oakden, Ryan Oglesby, Angus Lau, Hatef Mehrabian, Chris Heyn, Aimee K.M. Chan, Hany Soliman, Arjun Sahgal, Greg J. Stanisz

**Affiliations:** 1 Department of Medical Imaging, 7938 University of Toronto, Toronto, ON, Canada; 2 71545 Physical Sciences, Sunnybrook Research Institute, Toronto, ON, Canada; 3 Department of Medical Biophysics, 7938 University of Toronto, Toronto, ON, Canada; 4 Department of Radiation Oncology, 7938 University of Toronto, Toronto, ON, Canada

**Keywords:** chemical exchange saturation transfer, neuro-oncology, glioma, metastasis, progression, necrosis

## Abstract

Chemical exchange saturation transfer (CEST) is a relatively novel magnetic resonance imaging (MRI) technique with an image contrast designed for in vivo measurement of certain endogenous molecules with protons that are exchangeable with water protons, such as amide proton transfer commonly used for neuro-oncology applications. Recent technological advances have made it feasible to implement CEST on clinical grade scanners within practical acquisition times, creating new opportunities to integrate CEST in clinical workflow. In addition, the majority of CEST applications used in neuro-oncology are performed without the use gadolinium-based contrast agents which are another appealing feature of this technique. This review is written for clinicians involved in neuro-oncologic care (nonphysicists) as the target audience explaining what they need to know as CEST makes its way into practice. The purpose of this article is to (1) review the basic physics and technical principles of CEST MRI, and (2) review the practical applications of CEST in neuro-oncology.

## Introduction

The basis of chemical exchange saturation transfer (CEST) was first described in 1963,^1^ but it was not until 2000 when the results of the first CEST magnetic resonance (MR) experiment were published.^2^ Since then, a significant number of preclinical and clinical studies have demonstrated the potential utility of CEST MR imaging (MRI), alongside major technical developments, which have rendered it clinically feasible.^3^

The main advantage of CEST is its ability to measure in vivo metabolites of interest that may have low concentrations and can alter during different disease processes. Although some CEST applications use exogenous contrast agents^4,5^ and represent very relevant applications of CEST, many CEST applications in neuro-oncologic imaging do not require exogenous contrast agents such as gadolinium-based contrast agents.

Owing to higher spatial resolution and improved sensitivity to certain low-concentration metabolites compared to proton MR spectroscopy (MRS) methods,^6^ there has been a dramatic increase in new and emerging CEST methods in the recent decade and growing clinical translation of these methods, particularly for neuro-oncological applications.^3^ The purpose of this paper is to provide an overview that is accessible to a clinical neuro-oncological audience and to briefly summarize recent research in areas of unmet need in neuro-oncology.

## Basic Physics of CEST

### Chemical Shift

Hydrogen nuclei (or simply, “protons”) bonded to metabolites have a different precession frequency than those protons in water molecules, because of changes in the local magnetic field due to electron shielding. This phenomenon, referred to as chemical shift (typically defined in parts per million (ppm) or in Hertz, both relative to water in the case of CEST) is what enables techniques such as MRS and CEST to differentiate the signals coming from protons of different metabolites.

### Chemical Exchange

CEST imaging also relies on another intrinsic molecular phenomena called chemical exchange, whereby certain protons (eg, within metabolites) can physically switch places with another proton (eg, from nearby water molecules). Exchange is always happening and can occur with rates as high as hundreds or thousands of times per second. Using standard MRI methods, it can be difficult (if not impossible) to directly measure the signal from metabolites due to very low concentrations. However, the continuous exchange of protons with those of water molecules will thereby influence the water signal, which is detectable by standard MRI methods, making it possible to indirectly detect the metabolites.

**Figure 1.**
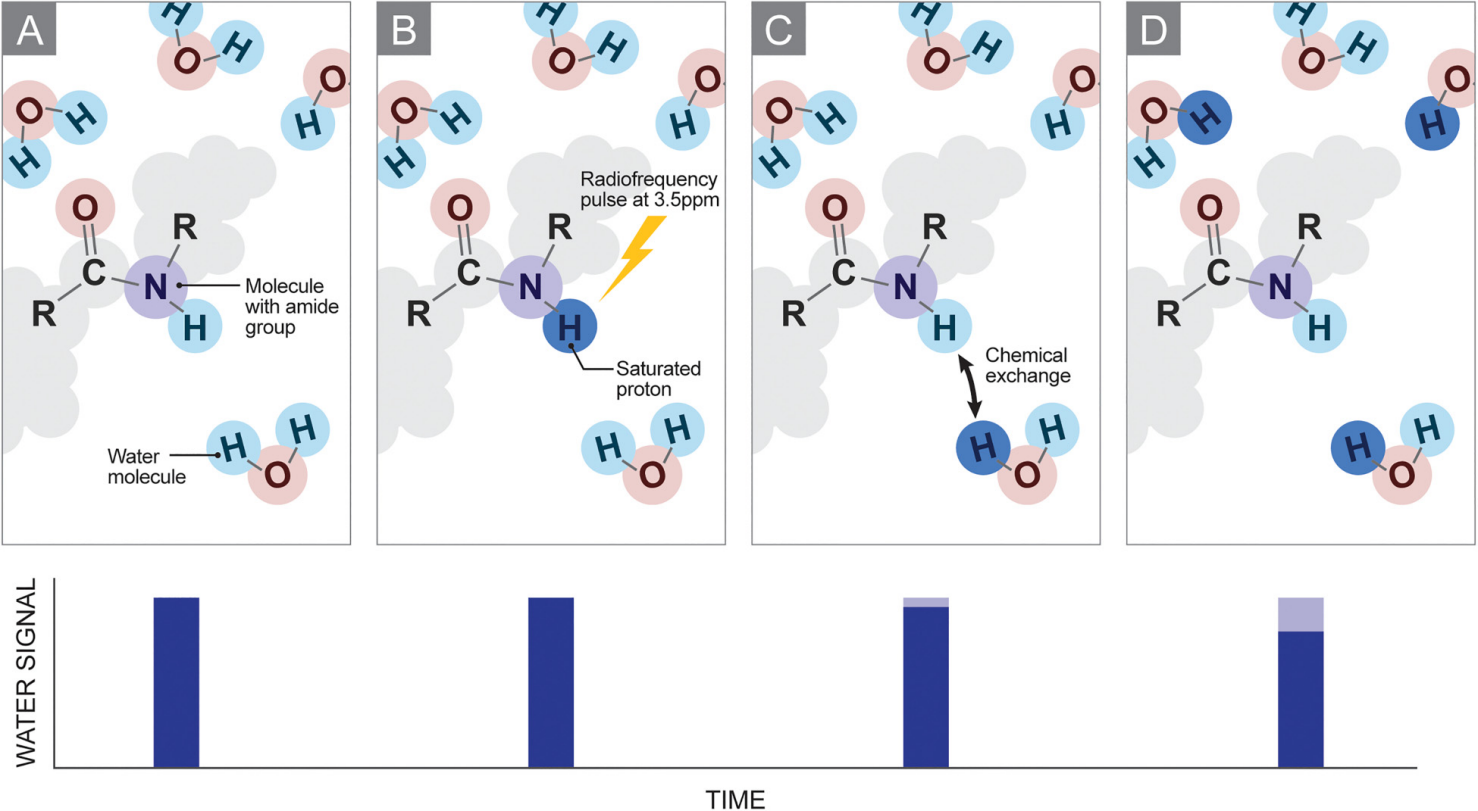
Schematic representation of chemical exchange saturation transfer (CEST): (A-D) Diagrams of the metabolite of interest containing an amide group are shown with surrounding water molecules, for each step in the CEST saturation and exchange process. The water signal level corresponding to each subfigure is shown below each panel. (A) Before saturation. (B) Radiofrequency saturation pulse is applied at a frequency offset of 3.5 ppm relative to water to saturate the proton in the amide group. (C) The saturated proton in the amide group is exchanged with the unsaturated water protons. (D) A continuous exchange of saturated protons leads to a measurable decrease in signal from water.

The first step of a CEST experiment is to use a long radiofrequency (RF) saturation pulse, or pulse train (on the order of seconds), applied at the resonance frequency of protons in the metabolite of interest to “saturate” them. The saturated protons will undergo chemical exchange (ie, physically switch places) with nonsaturated water protons. Immediately following this exchange, the MRI signal detected at the water frequency will decrease, due to the presence of protons that are already saturated (Figure 1). The difference in signal strength with and without the saturation pulse provides an indirect measure of the concentration the chemical group of interest, which could include amide, amine, guanidinium, or hydroxyl groups.

With sufficient time of RF saturation, a saturation steady state can be achieved providing maximal CEST contrast for the given pulse train. However, reaching a steady state is not always feasible as the duration of the RF pulse is limited by acquisition time, hardware constraints and specific absorption rate (SAR) limitations. As a result, the CEST contrast depends on numerous factors including the concentration of the metabolite of interest, efficiency of saturation, and exchange rate, which itself is dependent on both temperature and pH.^7^

Saturation pulse amplitudes and application duration are important factors for CEST imaging.^8,9^ Lower pulse amplitudes and longer durations (≥1 s) are used to select chemical groups with slower exchange rate and higher amplitudes and shorter durations for groups with faster exchange rate.^10^ The optimal amplitude also depends on the static field strength (*B*0). The consensus for amide proton transfer (APT) weighted brain imaging at 3T is 2 µT for 2 s.^3^ For the amide CEST peak, which has slower exchange, 1 to 3 µT allows for efficient labelling.^3^ For amine, guanidinium, and hydroxyl peaks, which have faster exchange, 2.9 to 12 µT have been used.^10^

### Measurement of CEST Signals

As stated above, the CEST contrast depends on the difference in the water signal between images acquired with and without RF saturation pulses at a specific frequency offset. However, there are several other mechanisms that affect the water signal during the saturation process.^11^ The two main considerations that need to be taken into account when measuring the CEST signal are magnetization transfer contrast (MTC) and the direct effect of water saturation.

MTC has been used in neuroimaging for several decades and refers to the transfer of magnetization between water and immobile semisolid macromolecules or myelin lipid content such as galactocerebrosides.^12^ These semisolid compounds have T2 relaxation times that are too short to be directly measured, while metabolites interrogated by CEST have much longer T2 relaxation times. The exchange rate of saturated protons with water protons is generally, but not always, faster in CEST (≈ 30-5500 Hz)^13^ than in MTC (≈ 20-40 Hz).^14^ Unlike CEST which is specific to the resonance frequency of protons in a particular metabolite, MTC occurs over a wide range of frequencies. As a result, MTC is less affected by the specific frequency offset.^7^

The direct effect of water saturation relates to the undesired saturation of water protons when intending to saturate protons in a metabolite of interest. This happens because an RF pulse has a nonzero width, so although it is centered on the resonance frequency of the proton of interest, it will directly saturate some water molecule protons. Measuring water signal when saturating protons at the opposite frequency offset (−Δ*ω*) can eliminate this and is referred to as the magnetization transfer ratio (MTR) asymmetry (MTR_asym_). Both the MTR and MTR_asym_ are the most commonly used parameters in CEST are defined as the following:

$$\mathrm{MTR}=\;\frac{S_{0}-S_{\mathrm{sat}}(\Delta \omega )}{S_{0}}$$

$$\mathrm{MT}R_{\mathrm{asym}}=\;\frac{S_{\mathrm{sat}}(-\Delta \omega )-S_{\mathrm{sat}}(\Delta \omega )}{S_{0}}$$

where *S*_sat_(−Δ*ω*) is the measured water signal with RF saturation at frequency offset −Δ*ω*, *S*_sat_(Δ*ω*) with RF saturation at Δ*ω* and *S*_0_ without RF saturation. This means that to calculate MTR_asym_, measurements at both Δ*ω* and −Δ*ω* are needed, assuming MTC and direct water saturation effects are symmetric and that the magnetic field (and therefore water frequency) is both uniform and constant. This assumption is true only to some extent, which will be discussed further. MTR consists of a mix of signal contributions, that is, from MT, CEST, and the direct effect, but it has higher signal-to-noise ratio compared to MTR_asym_ because MTR_asym_ propagates the error from two *S*_sat_ measurements. MTR can be a useful parameter in addition to more quantitative parameters that attempt to eliminate the T1 effects. For example, MTR maps have been shown to provide differentiation between tumor progression and radiation necrosis in brain metastasis patients.^15,16^

**Figure 2.**
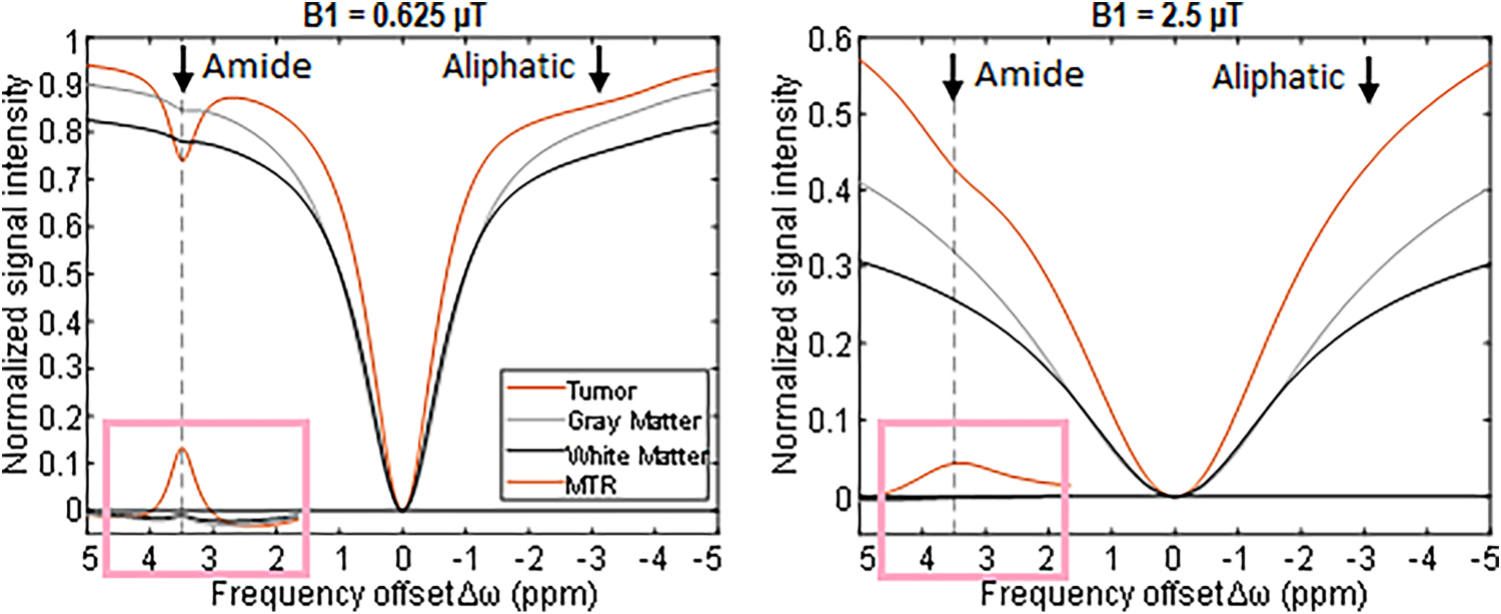
Simulated Z-spectra, with 2 different radiofrequency pulse amplitudes (B1) of 0.625 and 2.5 μT at 3T. The water resonance is defined to be 0 ppm. The amide (CEST) peak and center of the broad aliphatic (rNOE) peak are indicated. MTR asymmetry curve is shown in the left lower corner of each Z-spectra plot.

Abbreviations: CEST, chemical exchange saturation transfer; rNOE, relayed nuclear Overhauser effect; MTR, magnetization transfer ratio.

To describe and analyze the frequency offset-dependent saturation effects in CEST, an understanding of two commonly used plots is essential (Figure 2). The first plot is the CEST Z-spectrum. The horizontal axis is the frequency offset with the resonance of water at 0 ppm and the vertical axis is the ratio of water signal with saturation (*S*_sat_) to water signal without saturation (*S*_0_) at each given frequency offset. The CEST effects can be seen at the specific saturation frequency offsets of interest. As expected, the lowest point of the curve is at 0 ppm due to the direct saturation of water. The second plot (lower left corners in Figure 2) is the MTR asymmetry spectrum, where the signal at the opposite frequency offset has been subtracted to remove the effects of MTC and direct water saturation. However, a typical CEST experiment would collect images at only a few offsets, rather than measuring the entire Z-spectrum.

Another technique that has been used to remove some of the direct effect of water saturation is called Lorentzian difference (LD) analysis. This technique fits a Lorentzian function to the points on a Z-spectrum with minimal CEST effects to estimate the direct effect at all frequency offsets. Measurements typically fitted include those at high (|Δ*ω*| > 10 ppm^17^ or ≥ 6 ppm^18,19^) and low (|Δ*ω*| < 1.75 ppm^19^ or < 1 ppm^17,18^) frequency offsets. Then, the measured Z-spectrum values at the amide CEST frequency offset and its opposite (3.5 and −3.5 ppm, respectively) are subtracted from the Lorentzian values interpolated to those offsets to produce the LD signal. Furthermore, the measurements used to fit the Lorentzian likely have contributions from MTC unless the saturation amplitude is very low and therefore the fitted Lorentzian will only be an approximation of the signal from the direct effect.

### Factors Affecting the CEST Signal

**Figure 3.**
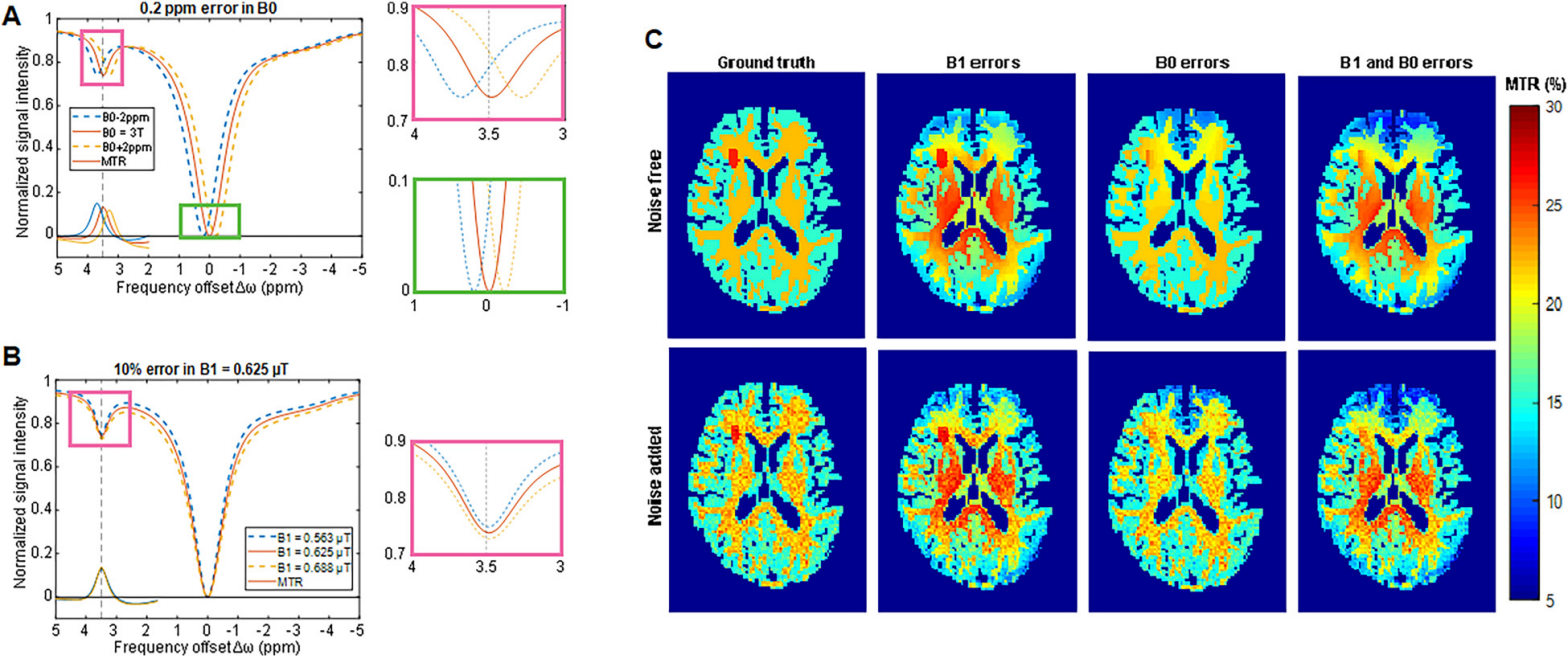
(A) Effect of B0 error as a shift to left (blue dotted line) or shift to right (orange dotted line) in the Z-spectrum and resultant underestimation of the APT signal. The solid red curve shows the corrected simulated spectrum. (B) Effect of B1 errors as a change in shape of the Z-spectrum. The corrected simulated spectrum is shown in red, the dotted curves demonstrate the effect of B1 errors, either 10% lower (blue) or 10% higher (orange) than prescribed, for a B1 of 0.625 μT. (C) Simulated MTR amide images of the brain at 3T (B1 = 0.625 μT) with gray and white matter, and a small simulated tumor in the right frontal region, showing the ground truth (left column), the effects of B1 and B0 errors both individually (middle columns), and combined (right column), in a noise-free simulation (top row) and a simulation with noise added (bottom row).

Abbreviations: MTR, magnetization transfer ratio; APT, amide proton transfer.

In order to accurately measure the CEST signal, the following need to be considered:

(a). **Magnetic field (B0) inhomogeneities:** CEST measurements are performed at one or more frequency offset(s). This assumes that the resonance frequency of water is at 0 ppm in each voxel. However, B0 inhomogeneities related to hardware and susceptibility changes related to patient anatomy (eg, near paranasal sinuses) create a shift in the B0 field, altering the actual frequency offset being saturated. This results in an apparent shift in the Z-spectrum along the *x*-axis (Figure 3). A commonly used correction technique densely and symmetrically samples spectral frequency at different offsets around the reference frequency. Techniques such as water saturation shift referencing (WASSR)^20^ or simultaneous mapping of water shift and B1 (WASABI)^21^ can then shift the Z-spectrum during postprocessing to recenter the water peak with the lowest point back to 0 ppm. Note that WASSR and WASABI scans should be performed separately from the CEST sequences. Alternatively, a much quicker way is to separately acquire a ΔB0 map to directly shift the Z-spectrum by the voxelwise ΔB0 value.^22^
(b). **RF field (B1) inhomogeneities:** The magnitude of the CEST effect is dependent on the saturation pulse(s) and, thus, an accurate amplitude B1 is required for quantification and reproducibility. However, RF inhomogeneity can cause variability in the amplitude of the RF pulse, resulting in an actual B1 amplitude that is different from the prescribed B1 amplitude. This can cause the CEST effect to be spatially inhomogeneous even in phantoms that have a known and uniform CEST effect.^23^ Furthermore, errors related to B1 inhomogeneities appear as a change in the shape of the Z-spectrum (Figure 3) and are generally nonlinear with respect to the frequency offset as well as applied B1 amplitude. These errors can be corrected in the same way as B0 inhomogeneities—by acquiring a B1 map and CEST data at several closely-spaced RF powers—however, these errors are more difficult to correct and require additional data such as B1 calibration maps, dual-transmit RF systems and more sophisticated data processing or model fitting using CEST scans collected with multiple nominal B1 amplitudes.^24^
(c). **Relayed nuclear Overhauser effect (rNOE): ** The rNOE effect has been attributed to aliphatic and olefinic protons^17,25^ in mobile macromolecules and is of unique importance,^26,27^ where it has been shown to decrease in tumors compared to healthy brain tissue.^17,28^ Effects from these chemical groups are evident as a broad peak in the Z-spectrum in the range of ≈ −1 to −4 ppm. rNOE is a multistep process. The first step is the saturation of a proton on the mobile macromolecule. The second step is through-space cross relaxation where the saturated magnetization is swapped with nonsaturated magnetization in an adjacent proton, such as on a hydroxyl group that is covalently bonded, or a water molecule that is hydrogen bound to the mobile macromolecule. Note that protons do not physically switch places as occurs in CEST. The third step is for the proton on the hydroxyl group to undergo CEST or the bound water molecule to be replaced with a free water molecule.^29^ The rNOE proton exchange rate is estimated to be around 16 Hz and the T2 to be at least an order of magnitude lower than that of the non-rNOE CEST exchange.^10,13^ The use of the rNOE-CEST effect for imaging is of unique importance, as exemplified by findings in glioma patients.^30–32^ Clinical application of CEST can be performed with the investigation of both APT and rNOE effects, especially as the NOE signal will affect the APT imaging. The presence of rNOE complicates CEST measurements, as the background signal is no longer symmetric. At high B1 power (˃ 2 µT), the contribution of magnetization transfer contrast (MTC) to the saturated CEST signal becomes greater than the effects of APT and NOE. However, the NOE effect cannot completely be eliminated.^13,33,34^ rNOE is itself an emerging MR biomarker in neuro-oncology.

### Practical Considerations

**a. Effect of field strength:** Increased field strength is advantageous to CEST as there is increased separation of signals, since the frequencies involved are much larger, and increased signal to noise ratio due to greater polarization. Another advantage is that a higher field strength leads to longer T1 relaxation time without affecting exchange rate, which increases the fraction of saturated protons and thus increasing CEST signal. Although CEST imaging is best at field strengths of 3T or higher, there have been CEST imaging performed at lower field strengths. This includes applications in glioma on 1.5T conventional scanner^35^ as well as the 1.5T MR-Linac, a hybrid MRI and radiation treatment device.^36^
**b. Contrast agents:** Although exogenous contrast agents have been used for CEST studies, most human CEST experiments have investigated signal from endogenous molecules with exchangeable protons in amide (3.5 ppm), amine (3 ppm), guanidinium (2 ppm), and hydroxyl groups (0.5-1.5 ppm).^10,13^ The most commonly studied CEST technique, APT, is focused on amide protons where the major source of amide is from dissolved proteins and peptides.^37,38^ Tumoral tissues can have increased concentration of proteins and peptides compared to normal brain making them an ideal candidate for APT imaging. As the CEST effect is dependent on T1 effects, CEST imaging should be performed before the administration of any contrast agent, including for example, gadolinium-based agents that reduce T1 relaxation time.
**c. Acquisition time:** Like any MRI acquisition, optimal CEST imaging is an interplay between acquisition time, image contrast and noise. In the past, most CEST experiments were limited to single-slice acquisitions, reducing its clinical applicability. Recently, significant efforts have been made to accelerate CEST imaging using both acquisition- and reconstruction-oriented approaches,^39^ enabling whole brain imaging with reasonable scan times. For example, a 3D APT protocol,^3^ where APT CEST are performed using 7 frequency offsets (at ±3.1, ±3.5, ±3.9 ppm with a reference scan) with concatenated RF pulses from 2 amplifiers and dixon-based ΔB0 mapping, is now able to produce a 10-slice volumetric image in 4 min and 8 s, with a resolution of 1.8 × 1.8 × 6 mm.^3^

   Long continuous RF pulses (∼5 s) are ideal for CEST but are not practical in clinical settings due to hardware, and more importantly SAR limitations. Various saturation pulses have been designed to improve the sensitivity and efficiency of CEST including pulse-train,^3^ pulsed steady-state,^40^ and unevenly segmented RF saturation.^41^ In addition, several innovative saturation editing sequences have been proposed.^42–46^ Fast readout sequences improve readout efficiency and decrease scan time. Turbo spin-echo is the most common readout approach used for CEST imaging on clinical scanners,^47^ as it does not suffer from susceptibility-related distortion and ghosting from lipids as seen with echo-planar-based sequences.^48^ Other readout sequences or alternative saturation approaches including ultrafast Z-spectroscopy^49^ can also be used to accelerate the acquisition.^34,50^ Parallel imaging,^51^ compressed sensing,^52,53^ or combination of both^54^ and keyhole imaging^55^ can also be used to speed-up acquisition. Other promising techniques include magnetic resonance fingerprinting^56^ and nonFourier transform-based techniques based on manifold learning.^57^

**d. Measurement reliability:** Reproducibility between vendors and sites is very important for clinical applicability of CEST, as it enables results from different cohorts to be compared.^58–60^ Generally, there is no standardization for CEST postprocessing. However, recent consensus recommendations have been published to address this issue.^3^ Studies have shown good repeatability in healthy subjects.^60,61^ In patients, repeatability is region-dependent, with better repeatability in supratentorial lesions compared to infratentorial lesions due to B0 inhomogeneity and susceptibility effects.^62^ In recent work,^63^ cross-vendor comparisons showed improved correspondence when the CEST signal was scaled by the contralateral normal-appearing white matter signal.

## Emerging Clinical Applications

### Differentiating Tumor Progression From Treatment Response

**Figure 4.**
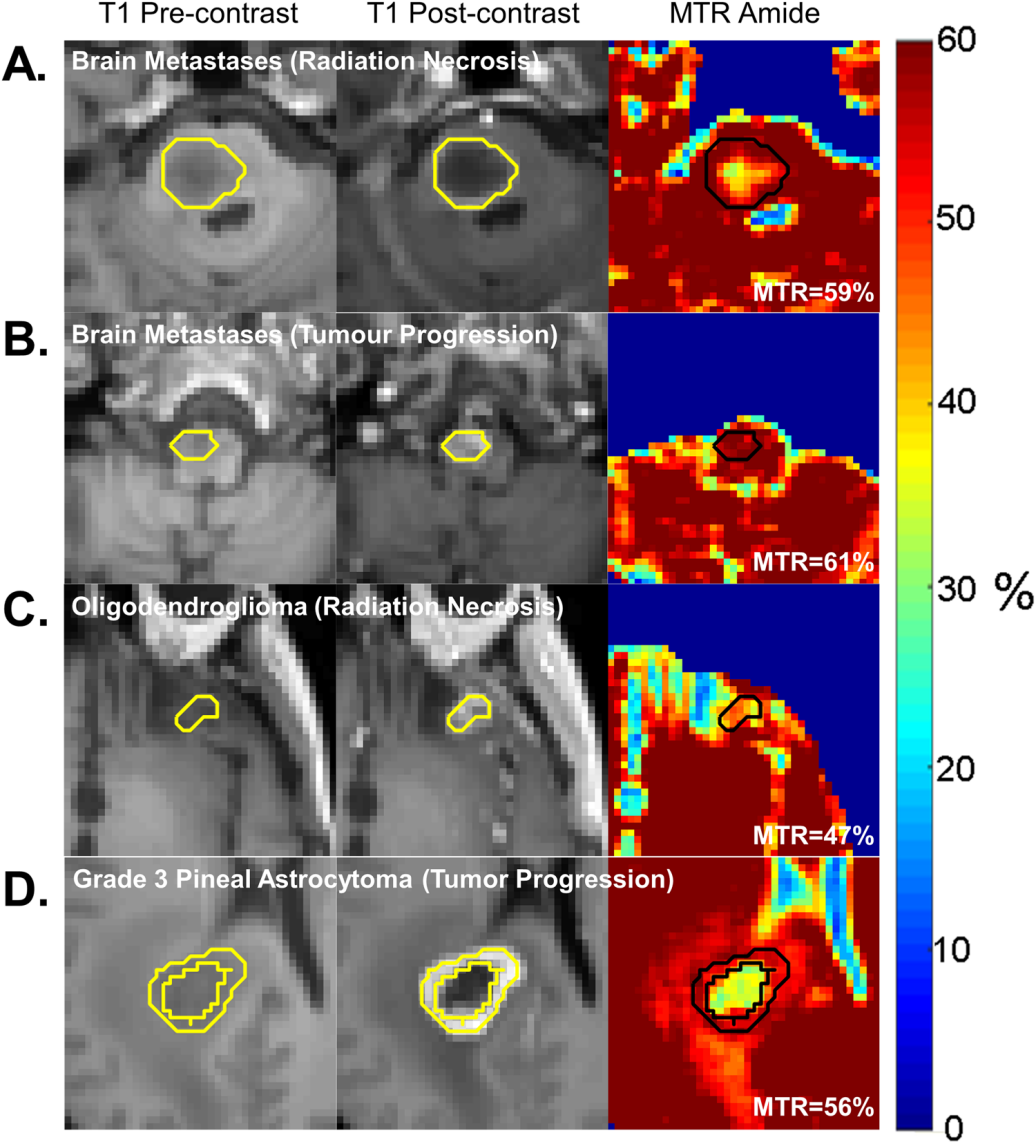
Chemical exchange saturation transfer (CEST) for differentiation of tumor progression versus radiation necrosis: examples of radiation necrosis (A) and tumor progression (B) are shown for patients with brain metastases following stereotactic radiosurgery. Examples of glioma are shown following chemoradiation, including an oligodendroglioma with radiation necrosis (C) and a progressed grade 3 astrocytoma (D). All images are of selected slices from the 3D CEST acquisition at 3T.

CEST, in particular APT CEST, can be added to the existing armamentarium of structural and physiologic imaging such as diffusion weighted imaging (DWI), perfusion weighted imaging (PWI), and MRS. In particular, CEST has particular applicability in differentiating between tumor progression and treatment response in metastatic brain disease treated with radiation, or in gliomas treated with chemoradiation. Tumor progression results in higher concentration of proteins and peptides with amide groups, resulting in higher signal compared to radiation necrosis, where cellular derived protein concentrations are low (Figure 4).^64^ Several studies have demonstrated that the addition of CEST to PWI and DWI metrics increases the diagnostic accuracy in differentiating tumor progression from treatment effects in patients with glioblastoma (AUC, 0.95-0.97 vs 0.84-0.91^65^ and AUC, 0.87 vs 0.92^66^). Studies to assess the additional benefit of CEST in the setting of metastatic brain disease are ongoing with a recent study showing CEST metrics were shown to differentiate radiation necrosis from tumor progression a group of 70 patients (75 lesions) with metastatic brain disease (AUC up to 0.88).^15^

### Evaluation of Treatment Response

**Figure 5.**
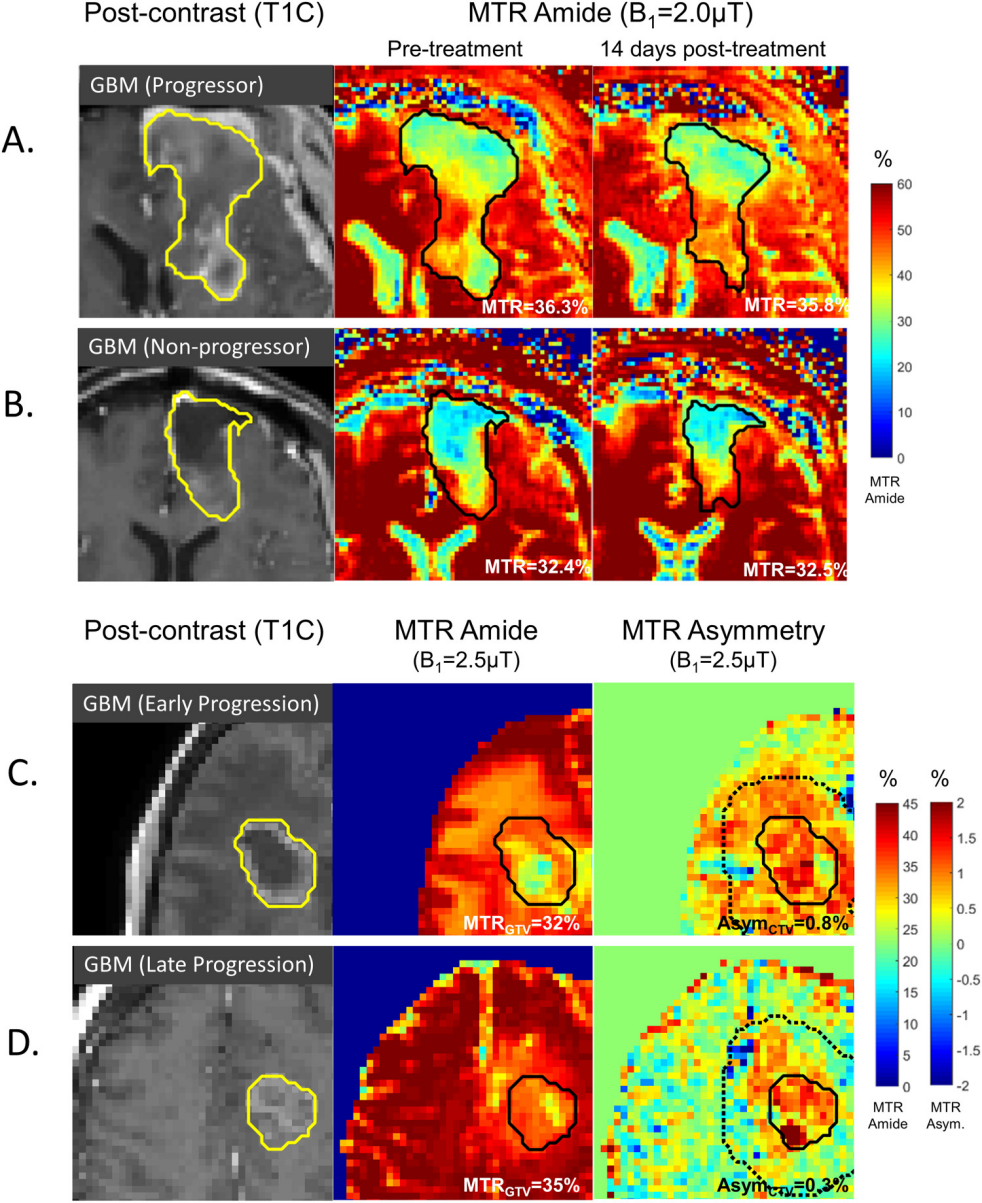
CEST for predicting treatment response in glioblastoma: (A, B) MTR amide maps from single-slice CEST at 3T are shown before treatment at the time of radiation planning MRI (middle column) and at 14 days after the start of radiation (right column) for patients demonstrating early progression in (A) versus late progression in (B). (C, D) Images at the time of radiation planning MRI are shown of the MTR amide map (middle column) and the MTR asymmetry map (right column). The solid lines include the enhancing tumor plus the resection cavity. The dotted lines on the MTR asymmetry maps represent the clinical target volume. Regions of high MTR asymmetry in the CTV were associated with early progression (C). An example of late progression (D) is also shown.

Abbreviations: CEST, chemical exchange saturation transfer; MTR, magnetization transfer ratio; MRI, magnetic resonance imaging; CTV, clinical target volume.

Studies conducted at 1.5 and 3T have demonstrated the potential to predict response to therapy in glioblastoma. Changes in MTR values between radiation planning MRI and 14 days following start of chemoradiation can identify patients who demonstrate early progression^67^ (Figure 5). High MTR values in the clinical target volume (CTV), defined as enhancing tumor and resection cavity with 2 to 3 cm margin which receives the highest radiation dose, before the start of chemoradiation are associated with early progression^36^ (Figure 5). In agreement with the known relationship between acidity and glioblastoma, a study of 20 patients with glioblastoma using amine CEST to determine pH demonstrated that patients with low acidity tumors have longer progression free survival compared to those with tumors showing stable or increased acidity postoperatively or throughout follow-up.^68^ Compared to glioblastoma, literature regarding CEST in brain metastasis is limited. In a group of 25 patients with brain metastasis undergoing stereotactic radiosurgery, CEST metrics in contralateral WM were strongly correlated with volume change of metastatic lesions at 1-month postradiation.^69^

### Other Applications

Clinical CEST applications include those that measure the APT effect for improved diagnosis of brain tumors, for example, in distinguishing brain metastases from glioblastoma,^71^ and also in differentiating lymphomas from high-grade gliomas,^72^ partially achieved by comparing the APT signal in areas of peritumoral edema. As well, CEST has been investigated to predict grading of the gliomas demonstrating higher APT signal in high versus low-grade glioma.^70^ However, most of the research studies in this area have been conducted before the release of new WHO classification of brain tumors which includes an array of new molecular and cytogenetic markers for tumor classification as opposed to purely microscopic features that were available at the time of these studies.^73^ CEST has been investigated to determine the molecular subtype of gliomas. Increased CEST effect in gliomas is observed with wild-type isocitrate dehydrogenase (IDH) gliomas compared to those with mutant IDH.^30,74^ Likewise, increased signal is observed in glioblastomas with unmethylated O6-methylguanine-DNA methyltransferase (MGMT) compared to their methylated counterparts.^75^ In addition, a positive correlation between CEST signal and tumor proliferation index (Ki-67 labeling) has been shown.^76^ Research in this area is still ongoing in light of new molecular biomarkers and technology.^73^ Besides APT CEST, amine CEST and GlucoCEST are examples of other applications that have also been explored in neuro-oncology.^77,78^

## Conclusion

In this article, basic principles of CEST were summarized for clinicians in neuro-oncological practice and several important areas were addressed where CEST MRI could provide added value to clinical care. This is an evolving field with many technical developments underway. For more detail, readers are encouraged to consult more in-depth publications^3,7,79,80^ and the references therein.

## Abbreviations

CEST: chemical exchange saturation transfer
RF: radiofrequency
SAR: specific absorption rate
APT: amide proton transfer
rNOE: relayed nuclear Overhauser effect
